# The Inhibitory Effect of Kakkonto, Japanese Traditional (Kampo) Medicine, on Brain Penetration of Oseltamivir Carboxylate in Mice with Reduced Blood-Brain Barrier Function

**DOI:** 10.1155/2015/917670

**Published:** 2015-02-19

**Authors:** Kousuke Ohara, Shinji Oshima, Nanami Fukuda, Yumiko Ochiai, Ayumi Maruyama, Aki Kanamuro, Akio Negishi, Seiichi Honma, Shigeru Ohshima, Masayuki Akimoto, Shingo Takenaka, Daisuke Kobayashi

**Affiliations:** ^1^Faculty of Pharmaceutical Sciences, Josai International University, 1 Gumyo, Togane, Chiba 283-8555, Japan; ^2^Faculty of Pharmaceutical Sciences, Josai University, 1-1 Keyakidai, Sakado, Saitama 350-0295, Japan; ^3^Onko-Do Kampo Akebono Yakkyoku Co., Ltd., 1-3-10 Gakuenhigashi-cho, Kodaira, Tokyo 187-0043, Japan

## Abstract

Oseltamivir phosphate (OP) is used to treat influenza virus infections. However, its use may result in central nervous system (CNS) adverse effects. In Japan, OP is used with Kampo formulations to improve clinical effectiveness. We evaluated the potential for using Kampo formulations to reduce CNS adverse effects by quantifying the CNS distribution of oseltamivir and its active metabolite oseltamivir carboxylate (OC) when administered with maoto and kakkonto. We administered lipopolysaccharide (LPS) by intraperitoneal injection to C57BL/6 mice to reduce blood-brain barrier function. Saline, maoto, and kakkonto were administered orally at the same time as LPS. OP was orally administered 4 hours after the last LPS injection and the migration of oseltamivir and OC was examined. Additionally, we examined the brain distribution of OC following intravenous administration. Changes in OC concentrations in the brain suggest that, in comparison to LPS-treated control mice, both Kampo formulations increased plasma levels of OC, thereby enhancing its therapeutic effect. Additionally, our findings suggest kakkonto may not only improve the therapeutic effect of oseltamivir but also reduce the risk of CNS-based adverse effects. Considering these findings, it should be noted that administration of kakkonto during periods of inflammation has led to increased OAT3 expression.

## 1. Introduction

Oseltamivir phosphate (OP) elicits antiviral effects by selectively inhibiting the neuraminidase in influenza viruses A and B, making it an effective agent for prevention and treatment of influenza infections. However, in 2012, Hoffman et al. reported a significant incidence of abnormal behavior and delirium induced by Tamiflu (odds ratios 29.35 and 13.50, resp.), based on the data collected in the Food and Drug Administration (FDA) Adverse Event Reporting System from 1999 to 2011 [1]. This study raised awareness of the adverse neuropsychiatric effects that may accompany the use of this anti-influenza agent. Based on the observation of a neuronal excitation effect following application of oseltamivir and its active metabolite oseltamivir carboxylate (OC) to juvenile rat hippocampal slices in vitro, Usami et al. reported that OP and OC may be involved in the induction of abnormal behavior [2].

Infections with the influenza virus are inflammatory diseases. Cytokines, such as tumor necrosis factor-alpha, interleukin-1 (IL-1), and IL-6, are secreted into serum and cerebrospinal fluid, with the increases in circulating levels of these cytokines resulting in attenuated blood-brain barrier (BBB) function [3, 4]. Reduced effectiveness of the BBB, which regulates the cerebral penetration of drugs, may increase the risk of adverse drug reactions, particularly in regard to drug-induced central nervous system (CNS) adverse effects, such as drowsiness, dizziness, and hallucinations [5]. In order to investigate these phenomena, brain distribution of OP and OC has been analyzed in juvenile rats, in which BBB function is underdeveloped [6, 7]. Additionally, animal models of inflammation have been used to analyze the distribution of drugs into the CNS. A number of approaches to creating these models of inflammation have been reported, including the induction of systemic inflammation through intraperitoneal (i.p.) administration of lipopolysaccharide (LPS) and the induction of local inflammation through intraplantar administration of Freund's complete adjuvant or carrageenan [8]. Systemic inflammation induced by LPS administration is reported to be pathologically similar to influenza-associated encephalopathy [4]. In our previous study, we assessed BBB integrity in mice with LPS-induced inflammation using extravasation of Evans blue (EB) dye as an indicator of BBB permeability. In addition to observed reduction in BBB function, we observed that oral administration of OP to these mice markedly increased the cerebral penetration of oseltamivir and OC [9].

The focus of integrative medicine is increasingly becoming the attainment of therapeutic effects through an organic fusion of the modern Western medicine, complementary therapies (such as biologic therapy, energy medicine, massage, and psychosomatic therapy), and alternative medicine approaches (such as traditional Chinese medicine and Ayurveda) [10]. In Japan, Kampo medicines, based on the traditional Chinese herbal medicine, is commonly used in a clinical setting. The Kampo formulation maoto is approved by the Ministry of Health, Labour, and Welfare as an insurance-covered treatment for influenza virus infections. OP is sometimes used concomitantly with maoto to achieve clinical effects [11, 12]. Maoto comprises four crude drug components: apricot kernel, ephedra herb, cinnamon bark, and* Glycyrrhiza*. In vitro studies have shown the ability of ephedra herb to inhibit the influenza virus uncoating [13], as well as the potential of cinnamon bark to inhibit membrane protein synthesis [14]. Additionally, the administration of maoto in a clinical setting was reported to achieve a general improvement of the influenza virus-associated condition and an antipyretic effect with effectiveness nearly identical to that of OP [12]. Furthermore, in comparison to OP monotherapy, concomitant treatment with maoto and OP is reported to effectively shorten the overall duration of fever and improve the general condition in terms of headaches and muscle pain, among other symptoms [11]. Kakkonto, a Kampo medicine currently not approved as an insurance-covered treatment, has been proposed for use as an adjunct to the treatment of influenza virus infections with OP, on the basis of the findings of an animal study [15] and its demonstration of effects similar to those reported with maoto in a clinical setting. Kakkonto comprises seven crude drug components: puerariae root, ephedra herb, cinnamon bark, peony root, ginger, jujube, and* Glycyrrhiza*. Among these components of kakkonto formulation, ephedra herb, cinnamon bark, and* Glycyrrhiza* are also present in maoto. In a study using mice infected with the influenza virus, kakkonto increased IL-12 production in bronchoalveolar lavage fluid in the initial stage of infection, inhibited body weight reduction, prolonged survival periods, and reduced the mortality rate [15].

Since adverse drug reactions (i.e., neuropsychiatric adverse effects) observed following OP administration are considered to result from the migration of oseltamivir or OC to the CNS, careful consideration must be given to the potential effects of the concomitant use of maoto or kakkonto on the distribution of oseltamivir and OC to the brain. Since no studies have directly evaluated this question thus far, we investigated the cerebral migration of oseltamivir and OC, which are involved in the onset of CNS adverse effects, when maoto or kakkonto is administered concomitantly with OP. Based on our findings, we report that inflammation-induced increase in brain penetration of OC was attenuated by the administration of kakkonto.

## 2. Materials and Methods

### 2.1. Drugs and Chemicals

OP and OC were purchased from Sequoia Research Products Ltd. (Pangbourne, UK) and MedChemexpress Co., Ltd. (Monmouth Junction, NJ, USA), respectively. Maoto (lot: F47711, J04981) and kakkonto (lot: F25972, J26252) extract granules were purchased from Tsumura Co., Ltd. (Tokyo, Japan). EB and LPS (derived from the* Salmonella enteric* serotype Typhimurium) were obtained from Sigma Chemical Co., Ltd. (St. Louis, MO, USA). The rabbit anti-*β*-actin polyclonal antibody (43 kDa), rat anti-MRP4 monoclonal antibody (150 kDa), and horseradish peroxidase- (HRP-) conjugated goat anti-rat immunoglobulin (IgG) were purchased from Santa Cruz Biotechnology, Inc. (Santa Cruz, CA, USA). The rabbit anti-OAT3 polyclonal antibody (59 kDa) was purchased from Biorbyt Ltd. (Cambridge, UK). HRP-conjugated goat anti-rabbit IgG was purchased from Enzo Life Sciences Inc. (Farmingdale, NY, USA). All other chemicals and solvents were of reagent or high-performance liquid chromatography (HPLC) grade and were used without further purification.

### 2.2. Animals

Male C57BL/6 mice (8 ± 1 weeks old) were purchased from Sankyo Labo Service Corporation, Inc. (Tokyo, Japan). Animals were reared under normal environmental conditions; lighting was maintained on a 12 h light-dark cycle, with ambient temperature maintained at 25 ± 2°C. All animal experiments were approved by the Institutional Animal Care and Use Committee of Josai University (approval number: H24011-2012/04/04).

### 2.3. Quantitative Quality Analysis of Two Kampo Formulations

Amounts of glycyrrhizic acid (GA), paeoniflorin (PA), and amygdalin (AM) in the formulations were measured as characteristic marker constituent compounds for quality control. GA (for Crude Drugs Determination), PA (for the Japanese Pharmacopoeia Crude Drugs Test), and AM (for the Japanese Pharmacopoeia Crude Drugs Test) were purchased as reference standards from Wako Pure Chemical Industries (Osaka, Japan). Samples of approximately 10 mg of maoto or kakkonto were measured in brown glass vials and 2 mL of 80% methanol and 1 mL of internal standard solution were added. Following 10 min of ultrasound irradiation, 10 min of shaking, and another 10 min of ultrasound irradiation, the solution was filtered through 0.45 *μ*m membrane filter (PTFE, Advantec, Tokyo, Japan). The amounts of each compound in the extract were measured using HPLC according to the peak height method. As shown in Table 1, the measurement conditions used were in accordance with the Japanese Pharmacopoeia, 16th Edition [16], and the method described by Honma et al. [17].

### 2.4. Animal Treatment for Induction of Inflammation

**Figure 1:**
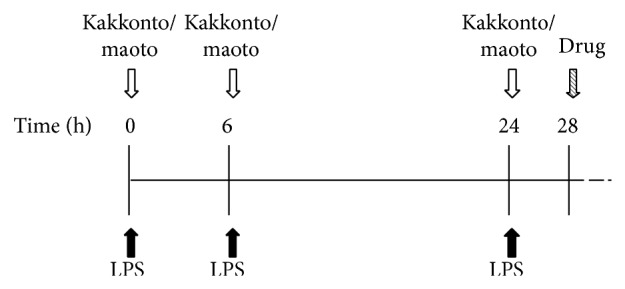
Experimental protocol for the animal study. Closed and open arrows indicate the intraperitoneal (i.p.) injections of lipopolysaccharide (LPS) and oral administration of Kampo formulations. Striped arrow indicates the administration of Evans blue (EB) dye or oseltamivir phosphate (OP). The concentrations of EB were determined 120 min after the intravenous injection. The concentrations of oseltamivir and oseltamivir carboxylate (OC) were determined at 5, 60, and 120 min following OP administration.

The experimental protocol of this study is schematically depicted in Figure 1. LPS (3 mg/kg in 0.2 mL saline/time) was administered by i.p. injections at 0, 6, and 24 h. Mice were orally administered maoto (Mao group) or kakkonto (Kak group), with both Kampo medications administered at 125 mg/kg in 0.1 mL saline at the time of LPS injections. The dose used was previously shown to be clinically relevant, corresponding to a human dose of 7.5 g/day for a 60 kg person. Saline (0.1 mL) was orally administered at the same intervals to the control animals (Con group).

### 2.5. Measurement of Plasma and Cerebral Concentrations of EB after Intravenous (i.v.) Administration

The integrity of BBB was evaluated by measuring the amount of EB extracted from brain tissue using our previously reported methods [9]. Briefly, 2% EB solution (4 mL/kg in saline) was injected via tail vein 4 h after the third injection of LPS. Mice were anesthetized with pentobarbital (100 mg/kg i.p.) and a blood sample was collected from the jugular vein. The thorax was opened and the right atrium was dissected out. Mice were perfused with 5 units/mL of heparinized saline (37°C) through the left ventricle until colorless perfusion fluid was obtained. After decapitation, the brain was rapidly excised and rinsed with ice-cold phosphate buffered saline (PBS, pH 7.4).

For quantitative determination of EB concentration, brain tissues were homogenized in 3.5 mL 50% trichloroacetic acid (TCA)/g tissue using a glass/Potter-Elvehjem tissue grinder (200 rpm, 2 min) with polytetrafluoroethylene (PTFE) in ice bath and centrifuged at 4°C (10,000 ×g, 15 min). The absorbance of supernatant was measured at 610 nm using a UV-2400 PC spectrophotometer (Shimadzu Corp., Kyoto, Japan). Blood samples were centrifuged at 4°C (2,000 ×g, 15 min) to obtain plasma. A sample of plasma (40 *μ*L) was mixed with the same volume of TCA and centrifuged at 4°C (12,000 ×g, 5 min). The absorbance of supernatant was measured at 610 nm.

### 2.6. Measurement of Plasma and Cerebral Concentrations of Oseltamivir and OC after Oral Administration of OP

OP (300 mg/kg in 0.2 mL saline) was orally administered 4 h after the third injection of LPS. Mice were anesthetized with pentobarbital and a blood sample was collected from the jugular vein at 5, 60, and 120 min after OP administration. The thorax was opened, and heparinized saline was perfused through the left ventricle until colorless perfusion fluid was obtained. The brain tissue of mice was rapidly removed after the decapitation and rinsed in ice-cold PBS. Three to 9 mice were used at each sampling time point in each group.

Oseltamivir and OC levels in plasma and brain tissues were quantified by HPLC, as described in our previous study [9]. Blood samples collected from the jugular vein were centrifuged to obtain plasma at 4°C (2,000 ×g, 15 min). Brain tissue samples were homogenized in 3.5 mL PBS/g tissue and centrifuged at 4°C (12,000 ×g, 10 min). Oseltamivir and OC were extracted from the resulting supernatant by solid-phase extraction (SPE) using Oasis MCX extraction cartridges (1 cc/30 mg, Waters, Milford, MA, USA) conditioned with 1 mL of methanol followed by 1 mL water. The supernatant sample (1 mL) was drawn through the cartridge and the cartridge was washed with 1 mL of 2% formic acid/water, followed by methanol and 0.005% ammonium hydroxide/methanol. The analytes were eluted with 3 mL of 5% ammonium hydroxide/methanol and evaporated to dryness under nitrogen at 40°C. The dried extracts were stored at −45°C until HPLC analysis.

The HPLC system consisted of a pump (LC-10AD_vp_, Shimadzu Corp., Kyoto, Japan), degasser (DGU-12A, Shimadzu Corp.), column (Zorbax SB-CN 5 *μ*m particle size, 4.6 × 250 mm, Agilent Technologies, CA, USA), column oven (CTO-10A, Shimadzu Corp.), and UV absorbance detector (SPD-10AV, Shimadzu Corp.). A mobile phase consisting of acetonitrile : 10 mM potassium dihydrogen phosphate buffer (pH 3.0) = 1 : 9 (v : v) was used for the elution at the flow rate of 1.5 mL/min. The column temperature was 50°C and the detector was operated at absorbance wavelength of 215 nm. The dried extract was reconstituted in 200 *μ*L of PBS and 40 *μ*L of the solution was injected onto the HPLC system.

### 2.7. Measurement of Carboxylesterase (CES) Activity

Liver microsomal fractions prepared from mice of Con, Mao, or Kak group were used as sources of the CES. Microsomal fractions were prepared as described previously [18].

For isolation of microsomal fractions, mice were perfused with 1.15% potassium chloride solution through the left ventricle and the liver was harvested 4 h after the third i.p. injection of LPS. The dissected liver tissue was washed in ice-cold PBS and homogenized with a 3 × volume of ice-cold PBS. The liver tissue homogenate was centrifuged at 4°C (10,000 ×g, 20 min). The isolated supernatant was subsequently centrifuged to achieve complete sedimentation of the microsomes (4°C, 105,000 ×g, 60 min). The pellet was washed by resuspension in 3 mL of homogenization solution and then resedimented at 105,000 ×g for 60 min. Total protein concentration in the microsomes was determined using a bicinchoninic acid (BCA) protein assay kit (Pierce, Rockford, IL, USA). The conversion of oseltamivir to OC was quantified by measuring OC concentration at 37°C in an incubation mixture containing the microsomal fraction in PBS (1 mg/mL) and 100 *μ*M oseltamivir over 60 min.

### 2.8. Measurement of Biochemical Parameters

Measurement of blood biochemistry parameters was performed using a VetScan VS1 Chemistry Analyzer (Abaxis, Inc., Union city, CA, USA). Mice were anesthetized and a 0.2 mL sample of blood was collected from the jugular vein with a syringe containing lithium heparin. The samples of whole blood (0.1 mL) were transferred to the rotor and the analysis was performed according to manufacturer's instructions. Alkaline phosphatase activity (ALP), alanine aminotransferase activity (ALT), amylase activity (AMY), creatinine levels (CRE), blood urea nitrogen levels (BUN), total protein concentration (TP), albumin concentration (ALB), total bilirubin concentration (TIBIL), sodium concentration (Na), potassium concentration (K), calcium concentration (Ca), inorganic phosphate concentration (PHOS), and glucose concentration (GLU) were measured.

### 2.9. Measurement of Plasma and Cerebral Concentrations of OC after i.v. Administration

OC (20 mg/kg in 0.1 mL saline) was injected via tail vein 4 h after the third injection of LPS. Blood samples were withdrawn from the jugular vein at predetermined times over 120 min. Brain and plasma OC concentrations were measured by a slight modification of the HPLC methods described in Section 2.6 [9, 19]. Briefly, collected plasma (70 *μ*L) was mixed with 910 *μ*L of 0.1 M hydrochloride acid and 70 *μ*L of the internal standard solution (15 *μ*g/mL pipamperone dihydrochloride). Collected brain tissues were mixed with 4.0 mL of 0.1 M hydrochloride acid/g tissue and homogenized in ice bath. Brain tissue homogenates were centrifuged at 4°C (12,000 ×g, 10 min). The resulting supernatant (900 *μ*L) was mixed with 145 *μ*L of 0.1 M hydrochloric acid and 55 *μ*L of the internal standard solution (20 *μ*g/mL pipamperone dihydrochloride). Resulting mixture was subjected to SPE using Oasis MCX and the analytes were evaporated to dryness under nitrogen. Dried extracts were stored at −45°C until HPLC analysis.

HPLC system for measurement of OC levels consisted of a Mightysil RP-18 GP column (3 *μ*m particle size, 2.0 × 150 mm, Kanto Chemical, Tokyo, Japan) and a guard column (Mightysil RP-18 GP 5-2.0, 3 *μ*m particle size, Kanto Chemical). A mobile phase consisting of acetonitrile : 10 mM potassium dihydrogen phosphate buffer (pH 3.0) = 1 : 9 (v : v) was used for the elution, at a flow rate of 2.0 mL/min. The column temperature was 40°C and the UV detector was operated at absorbance wavelength of 215 nm. The dried extract was reconstituted with 200 *μ*L of eluent and an aliquot (40 *μ*L) of the solution was injected onto the HPLC system.

### 2.10. Western Blot Analysis

Obtained brain tissue was homogenized with a 5 × volume of homogenate buffer (0.1 M Tris-HCl buffer, pH 8.0) containing 1 mM phenylmethylsulfonyl fluoride, 1 mM dithiothreitol, 20 *μ*g/mL aprotinin, leupeptin, and pepstatin in ice bath. Brain tissue homogenates were subsequently centrifuged at 4°C (6,500 ×g, 15 min). Isolated supernatants were subsequently centrifuged at 140,000 ×g for 30 min at 4°C. The pellet was dissolved in homogenate buffer containing 1% NP-40.

The protein concentration in the solution was determined using the BCA protein assay kit. Sodium dodecyl sulfate (SDS, 1%) was added to each sample and heated to 95°C. Protein solution (25 *μ*g per lane) was loaded onto a 7.5% SDS-polyacrylamide gel and subjected to electrophoresis. The separated proteins were transferred onto a nitrocellulose membrane. The membrane was blocked with PBS containing 0.1% Tween 20 (PBST) and 5% skim milk and then incubated at room temperature for 1 h. After washing 3 times with PBST (5 min for each wash), membranes were incubated at 4°C overnight with primary antibodies: anti-OAT3 antibody (diluted 1 : 500 with PBST), anti-MRP4 antibody (diluted 1 : 100 with PBST), or anti-*β*-actin antibody (diluted 1 : 1,000 with PBST). After washing 3 times with PBST, membranes were incubated at room temperature for 1 h with secondary antibodies (HRP-conjugated goat anti-IgG; 1 : 5,000). The membranes were washed 4 times with PBST and the bands were detected using the SuperSignal West Dura Extended Dura Trial Kit (Takara Bio. Inc., Shiga, Japan). The chemiluminescence was detected using an LAS-1000 instrument (Fujifilm, Tokyo, Japan). Pixel density of transporter proteins was determined using the Image J software program.

### 2.11. Statistical Analysis

All data are presented as means ± SD. Significance between group means was determined by the Tukey-Kramer test. *P* < 0.05 was considered to be significant. All analyses were conducted using software from the R Project for Statistical Computing (R version 2. 15.2).

## 3. Results

### 3.1. Amounts of Marker Constituent Compounds in Kampo Formulations

The amounts of marker constituent compounds in kakkonto and maoto formulations used in the present study were within the range listed in the Japanese Pharmacopoeia, 16th Edition (Table 2).

### 3.2. Effect of Maoto or Kakkonto Administration on BBB Integrity

**Figure 2:**
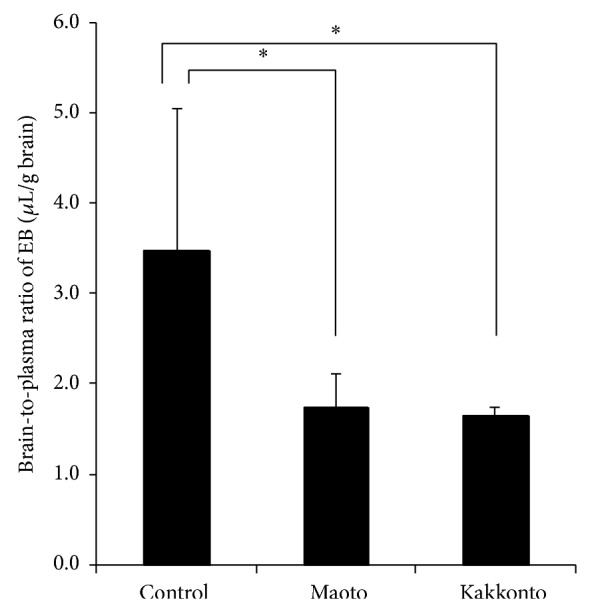
Effect of maoto or kakkonto administration on BBB integrity in mice with lipopolysaccharide- (LPS-) induced inflammation. Brain and plasma concentrations of Evans blue (EB) dye were determined 120 min after intravenous injection of EB. Data represent the means ± S.D. of 4–7 mice. ^*^ *P* < 0.05, Tukey-Kramer test.

The brain-to-plasma ratio (BPR) of EB, used as an index of BBB disruption, was measured in each treatment group, with the results presented in Figure 2. BPR in the Mao and Kak groups was 0.50- and 0.48-fold lower than those calculated in the Con group, respectively.

### 3.3. Plasma and Brain Concentrations of Oseltamivir and OC after Oral Administration of OP in Combination with Maoto or Kakkonto

**Figure 3:**
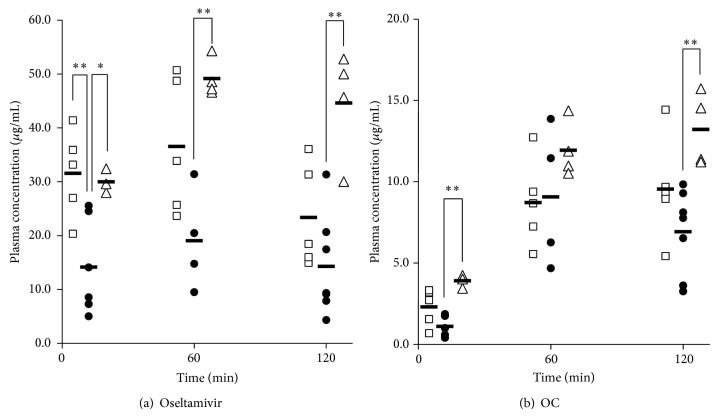
Plasma concentrations versus time profiles of (a) oseltamivir and (b) oseltamivir carboxylate (OC) after the administration of oseltamivir phosphate (OP) to mice with LPS-induced inflammation. Mice were pretreated with three i.p. injections of LPS (3 mg/kg) with concurrent oral administration of (●) saline (control), (□) maoto, or (△) kakkonto. A single oral dose of OP (300 mg/kg) was administered to mice 120 min after the third injection of LPS. Horizontal bar represents the mean values. ^*^ *P* < 0.05 and ^**^ *P* < 0.01, Tukey-Kramer test.

OP solution was orally administered to the mice in Con, Mao, and Kak groups. Figures 3(a) and 3(b) show the plasma concentrations of oseltamivir and OC following oral administration of OP. Plasma concentration of oseltamivir was increased in the Mao and Kak treatment groups during the experimental period compared to the Con group, with significant differences observed between the Kak and Mao groups. The area under the concentration time curve from 0 to 120 min following dose (AUC_0–120_) of the Mao group was 1.92 times higher than that of the Con group, whereas the AUC_plasma-oseltamivir, 0–120_ of Kak group was 2.60 times higher than that of the Con group. While no significant difference in plasma OC levels was observed between the Mao and Con groups, levels in the Kak group were significantly higher at 5 and 120 min after administration (Figure 3(b)). AUC_plasma-OC, 0–120_ of Mao and Kak groups was 1.12 and 1.57 times higher, respectively, than that of Con group.

**Figure 4:**
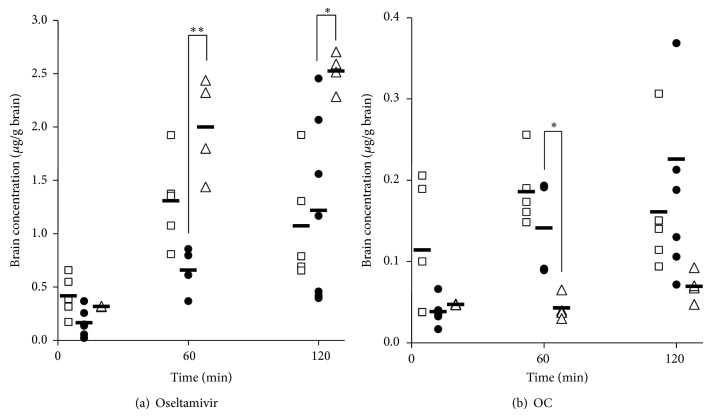
Brain concentrations versus time profiles of (a) oseltamivir and (b) oseltamivir carboxylate (OC) after the administration of oseltamivir phosphate (OP) to mice with lipopolysaccharide- (LPS-) induced inflammation. Mice were pretreated with three intraperitoneal (i.p.) injections of LPS (3 mg/kg) with concurrent oral administration of (●) saline (control), (□) maoto, or (△) kakkonto. A single oral dose of OP (300 mg/kg) was administered to mice 120 min after the third injection of LPS. Horizontal bar represents the mean values. ^*^ *P* < 0.05 and ^**^ *P* < 0.01 (versus control), Tukey-Kramer test.

Figures 4(a) and 4(b) show the cerebral concentrations of oseltamivir and OC following oral administration of OP. Cerebral oseltamivir levels were higher in the Kak group than in the Con group at 60 and 120 min after administration. While no significant difference in cerebral oseltamivir concentrations was noted during the experimental period in the Mao group, the levels were observed to be slightly higher than those measured in the Con group (Figure 4(a)). AUC_brain-oseltamivir, 0–120_ values in Mao and Kak groups were 1.51 and 2.52 times higher, respectively, than the mean AUC_brain-oseltamivir, 0–120_ measured in the Con group. Brain OC levels in the Kak group were lower than those measured in the Con group throughout the experimental period, reaching significant difference at 60 min after administration. AUC_brain-OC, 0–120_ values in Mao group were 1.18 times higher than those in the Con group, whereas those measured in the Kak group were 0.37 times lower than those measured in control mice.

The mean BPR was calculated as the ratio between mean AUC_brain_ and mean AUC_plasma_ and used as an index of the penetration of oseltamivir and OC into the brain. BPR of OC in the Kak group was significantly lower than that calculated in the Con group (Table 3). The cerebral migration of OC, which was increased by LPS injection, was inhibited by concomitant administration of kakkonto.

### 3.4. Evaluation of Esterase Enzymatic Activities

**Figure 5:**
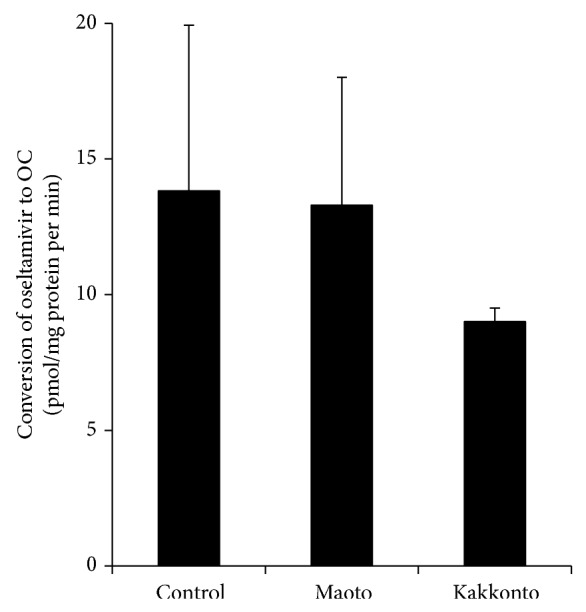
Effect of maoto or kakkonto Kampo formulations on the rate of oseltamivir conversion to the active carboxylate metabolite (OC) in mice with LPS-induced inflammation. Liver microsomal fractions prepared from mice administered three i.p. injections of 3.0 mg/kg LPS were used as a source of carboxylesterase. Data represent the means ± S.D. of 3–5 mice.

The conversion rates of oseltamivir to OC in liver microsomes from mice in Mao or Kak group were not significantly different from the conversion rate measured in the Con group (Figure 5).

### 3.5. Change in the Biochemical Markers by Maoto or Kakkonto Administration

The relevant biochemical parameters are summarized in Table 4. CRE concentration was decreased to the lower limit of quantification by the concomitant administration of kakkonto or maoto in LPS-treated mice. GLU concentration was significantly higher in Mao and Kak groups than in the Con group. No significant difference was observed in other biochemical parameters.

### 3.6. Plasma and Brain Concentrations of OC after Intravenous Administration of OC in Combination with Maoto or Kakkonto

**Figure 6:**
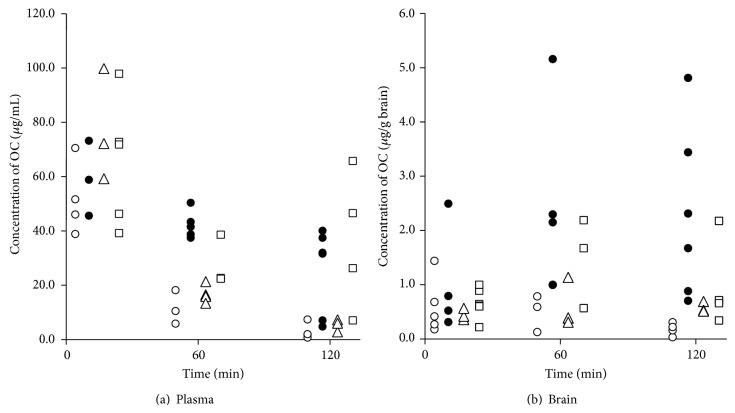
Plasma (a) and brain (b) concentrations versus time profiles of oseltamivir carboxylate (OC) after the administration of OC to mice with lipopolysaccharide- (LPS-) induced inflammation. Mice were pretreated with three intraperitoneal (i.p.) injections of LPS (3 mg/kg) with concurrent oral administration of (●) saline, (□) maoto, or (△) kakkonto. Control animals were administered saline vehicle without LPS (○). A single intravenous dose of OC (20 mg/kg) was administered to mice 120 min after the third injection of LPS.

Figure 6 shows the plasma (panel (a)) and cerebral (panel (b)) OC concentrations after intravenous administration of OC. In the kakkonto-treated mice, plasma OC levels were lower than those measured in LPS-treated control animals (Con group). No significant difference was observed between the Mao and Con groups in plasma OC levels. Moreover, cerebral OC levels in the mice of Kak group were lower than those of the Con group. While cerebral OC levels in the Mao group were lower than those measured in the Con group, they were not different from those measured in the Kak group. Plasma and cerebral OC levels in the LPS-injected mice were elevated compared to the corresponding levels in the mice that were not treated with LPS. Coadministration of kakkonto attenuated this increase in OC concentration and reduced the variation in OC levels. The difference in OC levels between Kak and Con groups was analyzed using the *F*-test at each time point, with the difference in the mean value between Kak and noninflammatory (without LPS administration) groups analyzed using the unpaired *t*-test at each time point. Comparing the Kak and Con groups, significant differences were observed in the brain OC concentrations throughout the experimental period and in the plasma at 120 min after administration (plasma: 120 min; *P* = 0.044, brain: 5 min; *P* = 0.023, 60 min; *P* = 0.036, 120 min; *P* = 0.008). Additionally, comparing the mean OC levels in Kak group with those measured in mice who did not receive LPS treatment, significant differences in cerebral concentrations were only found 120 min after administration, while no significant differences were observed in plasma concentration (plasma: 5 min; *P* = 0.106, 60 min; *P* = 0.204, 120 min; *P* = 0.304, brain: 5 min; *P* = 0.638, 60 min; *P* = 0.853, 120 min; *P* = 0.005). These results suggest that kakkonto suppresses the increased brain penetration of OC in the presence of inflammation.

### 3.7. Effect of Maoto or Kakkonto on the Expression of OAT3 and MRP4 in the Brain

**Figure 7:**
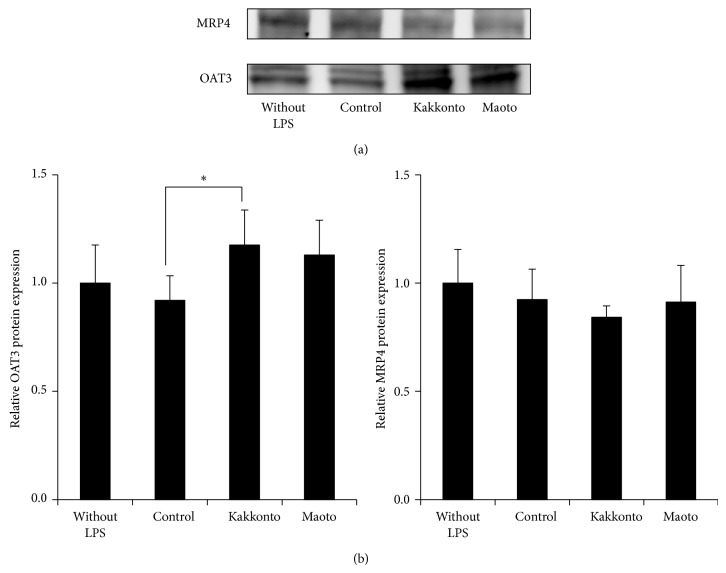
Effects of kakkonto and maoto on the expression of OAT3 and MRP4 proteins in the brain. (a) Representative images of the expression of OAT3 and MRP4 proteins in the brain and (b) OAT3 and MRP4 expression levels in the brain, quantified by Western blot analysis. Data represent the mean ± S.D. of 6 mice. ^*^ *P* < 0.05.

The results of Western blotting analysis demonstrated that brain OAT3 expression in the Kak group was 1.28 times higher than that in the Con group, with only Kak group showing an increase. While OAT3 expression was observed to be significantly increased following kakkonto administration, no changes in MRP4 expression were noted between the groups (Figure 7).

## 4. Discussion

In children with influenza virus infections, the use of OP is indicated to be associated with abnormal behavior. The concomitant use of OP and maoto in patients infected with the influenza virus was reported to be effective in significantly shortening fever duration, as well as achieving early improvement of physical symptoms such as fatigue, dizziness, and loss of appetite [11, 12]. In addition, kakkonto, which has a composition of crude drugs similar to that of maoto, has demonstrated in the results of a basic study [15] and in clinical settings the potential to achieve results similar to those of maoto. Despite the potential benefits of concomitant administration of kakkonto with OP, we found no studies evaluating the cerebral penetration of oseltamivir or OC with the use of these Kampo formulations.

The migration of substances from the blood to the cerebral parenchyma is regulated by the BBB. BBB is composed of tight junctions between the endothelial cells of brain microvasculature, astrocytes, and pericytes. Additionally, efflux transporters affect the penetration of substrates through the BBB. The substance permeation mechanism of the BBB, which is based on passive diffusion, is divided into the paracellular and the transcellular pathways [20]. Paracellular pathway is involved in the migration of water-soluble low-molecular weight (MW) compounds. However, because of the presence of tight junctions, almost no substance penetrates the BBB by this pathway. Conversely, transcellular pathway is involved in the penetration of lipid-soluble low-MW compounds into the brain. The penetration through this pathway, however, is restricted to compounds with MW of <500 or a log⁡*P* near 2. One method of assessing the integrity of BBB is to inject EB i.v. and subsequently measure the amount of EB that entered into the cerebral parenchyma. EB is a water-soluble dye with a MW of 960.8 that shows nearly complete binding to plasma albumin (MW of approximately 66,000). Therefore, it normally does not penetrate the BBB [21]. However, when BBB function is attenuated with cell injury as an inflammation, the paracellular pathway opens, and EB bound to albumin in the blood enters the cerebral parenchyma. Using the BPR of EB as an indicator, we have previously reported that BBB function is attenuated in mouse models of LPS-induced inflammation [9]. In the present study, oral administration of kakkonto and maoto formulations along with i.p. injection of LPS resulted in lower EB BPR, as compared to the BPR measured in animals treated with i.p. injections of LPS alone. This result demonstrates that the compromised integrity of the BBB caused by the LPS-induced inflammation was ameliorated by the administration of kakkonto and maoto. This observed improvement in BBB integrity may reflect a restoration of the barrier function blocking the paracellular brain penetration pathway.

Next, we evaluated the effect on brain penetration of oseltamivir and OC following oral administration of OP in a mouse model of inflammation. Due to their physical properties, oseltamivir (MW = 312.0, *c*log⁡*P* = 1.29) and its active metabolite OC (MW = 284.4, *c*log⁡*P* = −0.97) do not migrate easily by passive diffusion from the blood to the cerebral parenchyma [22, 23]. Additionally, oseltamivir is a substrate for the efflux transporter P-glycoprotein (P-gp), while OC is a substrate for the organic anion transporter (OAT) 3 and multidrug resistance protein (MRP) 4. Since oseltamivir and OC are excreted from the cerebral parenchyma to the blood, they normally do not distribute to the CNS [24].

In our previous report, with oral administration of OP in a noninflammation group (injected with saline vehicle) and a control group (injected with LPS in saline), the plasma concentration of oseltamivir was found to be markedly higher in the LPS-treated animals than in the noninflammation group, whereas plasma OC concentrations were similar. Additionally, brain concentrations of oseltamivir and OC were markedly higher in LPS-treated mice than in the saline-treated animals [9]. Pueraria root and peony root, crude components of the kakkonto formulation, promote gastrointestinal motility and dilate peripheral arteries, respectively, while cinnamon bark, a common crude drug component of both kakkonto and maoto, also dilates peripheral arteries [25]. The increase in plasma concentrations of oseltamivir in the present study may therefore be the result of increased absorption of oseltamivir from the intestine due to the promotion of gastrointestinal motility. Additionally, administration of kakkonto or maoto did not yield any change in CES activity in the liver in comparison to the control group. Therefore, changes in plasma OC concentrations may reflect increased plasma concentrations of the prodrug oseltamivir. Furthermore, considering brain concentrations of oseltamivir and OC from the perspective of the risk of CNS adverse effects, brain concentrations of oseltamivir in mice treated with Kampo formulations, and brain concentrations of OC in the maoto-treated mice demonstrated similar patterns to those observed in blood levels. Conversely, kakkonto-treated mice demonstrated differences in brain OC concentrations different from those observed in blood concentrations; namely, brain OC concentrations were found to be decreased in comparison to the control group, regardless of increased blood OC levels. Lower cerebral penetration (indexed as lower BPR) relative to the control group was observed only with OC levels in the kakkonto group. Overall, these findings suggest that kakkonto and maoto differ from each other in their effect on brain OC levels. In order to assess in more detail the effect of kakkonto on cerebral penetration of OC, we evaluated the changes in blood biochemistry and assessed the effect of i.v. administration of OC.

In our serum biochemical examination, oral administration of kakkonto or maoto decreased CRE concentration to the lower limit of quantification, suggesting that renal function returned to values within normal limits. Maoto and kakkonto are commonly used to treat colds and inflammatory diseases. Previously performed study reported an inhibition of carrageenan-induced edema and cotton pellet-induced granuloma formation in mice, suggesting that kakkonto and maoto inhibit the effusion phase and the late growth phase of the early stage of inflammation [26]. Our results may, therefore, signify that the markedly reduced renal function and somewhat reduced hepatic function observed in mice with BBB hypofunction can be recovered through the anti-inflammatory effects of kakkonto and maoto. Because OC is a renally excreted drug, lower plasma concentrations of OC (associated with the recovery of renal function) were noted after administration of maoto or kakkonto when compared with the LPS-treated Con group. Moreover, administration of kakkonto led to increased OAT3 expression in the brain compared to the expression in Con group, which may account for the decreased brain concentrations of OC.

The results of the present study show that maoto and kakkonto Kampo formulations increase plasma concentrations of oseltamivir and OC. Maoto and kakkonto exhibit different effects on OC distribution, with only kakkonto reducing the brain concentrations of OC. These findings indicate that the concomitant use of kakkonto with OP may reinforce the anti-influenza effect and reduce the risk of CNS adverse effects. Our results also suggest that the concomitant use of maoto with OP may reinforce the anti-influenza effect with no effect on the risk of CNS adverse effects. Further studies are warranted to evaluate the potential for applications of these two Kampo formulations as adjunct to OP treatment of influenza infection in a clinical setting.

## Figures and Tables

**Table 1:** The measurement conditions of maker compound for quality control in kakkonto and maoto formulations.

Maker compound for QC	Column	Column temperature (°C)	Mobile phase	Flow rate (mL/min)	Wavelength (nm)	Internal standard solution (final concentration)
GA	TSK-gel ODS-80TM (4.6 × 250 mm)	40	200 *μ*L/L phosphoric acid : acetonitrile (66 : 34)	1.5	254	30 *μ*g/mL amyl 4-hydroxybenzoate
PA			Deionized water : acetonitrile : phosphoric acid (850 : 150 : 1)	1.2	232	30 *μ*g/mL methyl 4-hydroxybenzoate
AM			50 mM potassium dihydrogen phosphate : methanol (5 : 1)	1.2	210	3 *μ*g/mL methyl 4-hydroxybenzoate

**Table 2:** Amounts of marker constituent compounds in kakkonto and maoto extract formulations.

Kampo formulation	Maker compound for QC	Amount listed in JP16 (in 7.5 g)	Amount per lot (in 7.5 g)	
			H44002	J26252

Kakkonto	Glycyrrhizic acid	19–57 mg	27.7 ± 0.2 mg	25.8 ± 0.36 mg
	Paeoniflorin	14–56 mg	27.7 ± 0.4 mg	30.4 ± 0.63 mg

			F47711	J04981

Maoto	Glycyrrhizic acid	14–42 mg	31.1 ± 0.46 mg	31.1 ± 0.25 mg
	Amygdalin	48–192 mg	102.7 ± 2.87 mg	90.4 ± 3.66 mg

Data represent the mean ± S.D. (*n* = 3).

**Table 3:** The AUC_0–120_ and the mean brain-to-plasma ratio (BPR) of oseltamivir and oseltamivir carboxylate (OC) following oral administration of oseltamivir phosphate (OP) to mice with lipopolysaccharide- (LPS-) induced inflammation.

Group	AUC				BPR	
	Plasma (min·*μ*g/mL)		Brain (min·*μ*g/g brain)		(*μ*L/g brain)	
	Oseltamivir	OC	Oseltamivir	OC	Oseltamivir	OC
Con	1949.86	762.01	79.35	16.06	40.70	21.08
Mao	3749.85	856.89	119.92	18.95	31.98	22.12
Kak	5066.10	1199.90	200.21	5.98	39.52	4.98

Con: control; Kak: kakkonto-treated; Mao: maoto-treated.

**Table 4:** Blood biochemistry parameters in mice with lipopolysaccharide- (LPS-) induced inflammation.

Parameter	(units)	Con	Mao	Kak
ALP	U/L	51.4 ± 47.96	25.5 ± 9.57	33.6 ± 7.57
ALT	U/L	71.2 ± 33.81	61.5 ± 14.29	69.6 ± 8.08
AMY	U/L	1058.2 ± 240.48	796.2 ± 63.24	949.8 ± 225.76
CRE	mg/dL	0.5 ± 0.24	<0.2^#,∗^	<0.2^#,∗^
BUN	mg/dL	75.8 ± 51.91	70.7 ± 24.71	59.4 ± 9.42
TP	g/dL	5.3 ± 0.23	5.0 ± 0.33	5.0 ± 0.13
ALB	g/dL	2.7 ± 0.17	2.7 ± 0.15	2.6 ± 0.14
TBIL	mg/dL	0.3 ± 0.04	0.2 ± 0.09	0.2 ± 0.05
Na+	mmol/L	145.8 ± 6.14	141.3 ± 3.67	139.4 ± 2.97
K+	mmol/L	6.9 ± 1.10	7.2 ± 1.39	7.3 ± 1.07
Ca++	mg/dL	9.4 ± 0.22	9.1 ± 0.45	8.9 ± 0.37
PHOS	mg/dL	10.2 ± 3.13	9.4 ± 1.73	10.2 ± 0.82
GLU	mg/dL	33.6 ± 19.81	69.3 ± 10.56^*^	65.4 ± 6.99^*^

Data represent the mean ± S.D. (*n* = 5-6).

^*^ *P* < 0.05 (versus control).

^#^Below the limit of quantitation.
